# Retraction of “The Chemistry Clinic: Authentic Assessment in a Student Led Environment”

**DOI:** 10.1021/acs.jchemed.5c00732

**Published:** 2025-06-20

**Authors:** Debra Willison

The author retracts this article (DOI: 10.1021/acs.jchemed.3c01319) following an institutional investigation by the University of Strathclyde’s Research Standards Advisory Group. This investigation “determined that research misconduct has taken place” due to reproduction of case studies and assessment forms contained within the Supporting Information from a previous publication (The Chemistry Clinic: Collaborative Teamwork to Achieve Innovative Solutions/DOI: 10.4018/978-1-6684-8198-1.ch008) without permission of the authors.

The original article was published on June 19, 2024 and was retracted on June 20, 2025.

